# A Novel Titanium Cranioplasty Technique of Marking the Coronal and Squamosoparietal Sutures in Three-Dimensional Titanium Mesh as Anatomical Positioning Markers to Increase the Surgical Accuracy and Reduce Postoperative Complications

**DOI:** 10.3389/fsurg.2021.754466

**Published:** 2021-12-14

**Authors:** Bing-Sen Xie, Fang-Yu Wang, Shu-Fa Zheng, Yuan-Xiang Lin, De-Zhi Kang, Wen-Hua Fang

**Affiliations:** Department of Neurosurgery, The First Affiliated Hospital of Fujian Medical University, Fuzhou, China

**Keywords:** cranioplasty, three-dimensional, titanium, surgery, accuracy, outcome

## Abstract

**Objective:** The objective of this research is to modify the titanium cranioplasty (Ti-CP) technique to increase the surgical accuracy and preliminarily verify the effectiveness and safety of this improvement.

**Methods:** We developed a novel technique of marking the coronal and squamosoparietal sutures in three-dimensional (3D) titanium mesh as anatomical positioning markers and designed a prospective trial in patients with a unilateral frontotemporoparietal skull defect. Patients were randomly divided into two groups by the presence or absence of the anatomical positioning markers, and the therapeutic effects of these two groups were compared.

**Results:** Forty-four patients were included in this study, including 28 (64%) males and 16 (36%) females. The mean age was 44.8 ± 15.2 years (range, 13–75 years). Overall postoperative complication rate of the intervention group (18%) was significantly (*P* = 0.03) lower than the control group (50%). Surgical accuracy of the intervention group (97.8%) was significantly (*P* < 0.001) higher than the control group (94%). Visual analog scale for cosmesis (VASC) of the intervention group (8.4) was significantly (*P* < 0.001) higher than the control group (7). The overall postoperative complication rate was 34%. Multivariate analyses showed that surgical accuracy <95.8% (OR = 19.20, 95% CI = 3.17–116.45, *P* = 0.001) was significantly associated with overall postoperative complications. Independent predictor of overall postoperative complications was surgical accuracy (OR = 0.57, 95% CI = 0.40–0.82, *P* = 0.002).

**Conclusions:** This novel technique for repairing frontotemporoparietal skull defects increases surgical accuracy, improves cosmetic prognosis, and reduces postoperative complications. Therefore, it is a safe and effective improvement for Ti-CP.

## Introduction

Cranioplasty (CP) is a well-known and frequent procedure in modern neurosurgery. Brain protection and cosmetic aspects are the major indications (1). Many different products ranging from autologous bone grafts to synthetic materials are used for CP (2). Autologous CP using the previously removed bone flap from the decompressive craniectomy (DC) is the first-line treatment for covering the large skull defect (3). However, both bacterial infections and absorption of the bone are common problems (4). Therefore, the use of prosthetic material is a standard practice in most hospitals in China (5). Titanium has become one of the most widely used prosthetic materials due to its chemical and biological stability (6). Moreover, it can be made symmetrical to the contralateral side of the skull and fit perfectly to skull bones of patients by using the three-dimensional (3D) titanium mesh (6).

Cranioplasty (CP) is regarded as a relatively simple and straightforward intervention (4, 7); however, it is associated with a high incidence of postoperative complications (8–11). Unlike other custom-made implants which are directly embedded in the defect of skull, titanium mesh is generally larger to cover the skull defect. Once the titanium mesh is placed, an appropriate adjustment is made to ensure precise position intraoperatively. However, titanium cranioplasty (Ti-CP) is performed without anatomical positioning markers, and the position of implant is determined subjectively. As a result, it is difficult to increase the surgical accuracy and decrease the risk of complications (12). This is a disadvantage of titanium mesh compared with other custom-made implants. Since no study has been reported to effectively solve this problem, we tried to modify the 3D Ti-CP technique to increase the surgical accuracy and preliminarily verify the effectiveness and safety of this improvement.

## Methods

### Development of a Novel Ti-CP Technique

The surgical accuracy of Ti-CP refers to the accuracy of the placement of titanium mesh. The more precise position of the titanium mesh, the higher accuracy. A simple and effective method to improve the surgical accuracy is to make anatomical positioning markers in the 3D titanium mesh so that the titanium mesh can objectively match the skull defect. As to anatomical positioning markers, the following conditions are required: (1) universality: anatomical positioning markers can be made in each titanium mesh for patients with a frontotemporoparietal skull defect after DC; (2) accuracy: anatomical positioning markers in titanium mesh must be objective and obvious, and the matching of the titanium mesh and the skull defect using the anatomical positioning marker must be precise; and (3) no additional iatrogenic injury: Ti-CP using anatomical positioning markers should be similar to traditional operations without additional iatrogenic injuries, such as increasing surgical risk.

**Figure 1:**
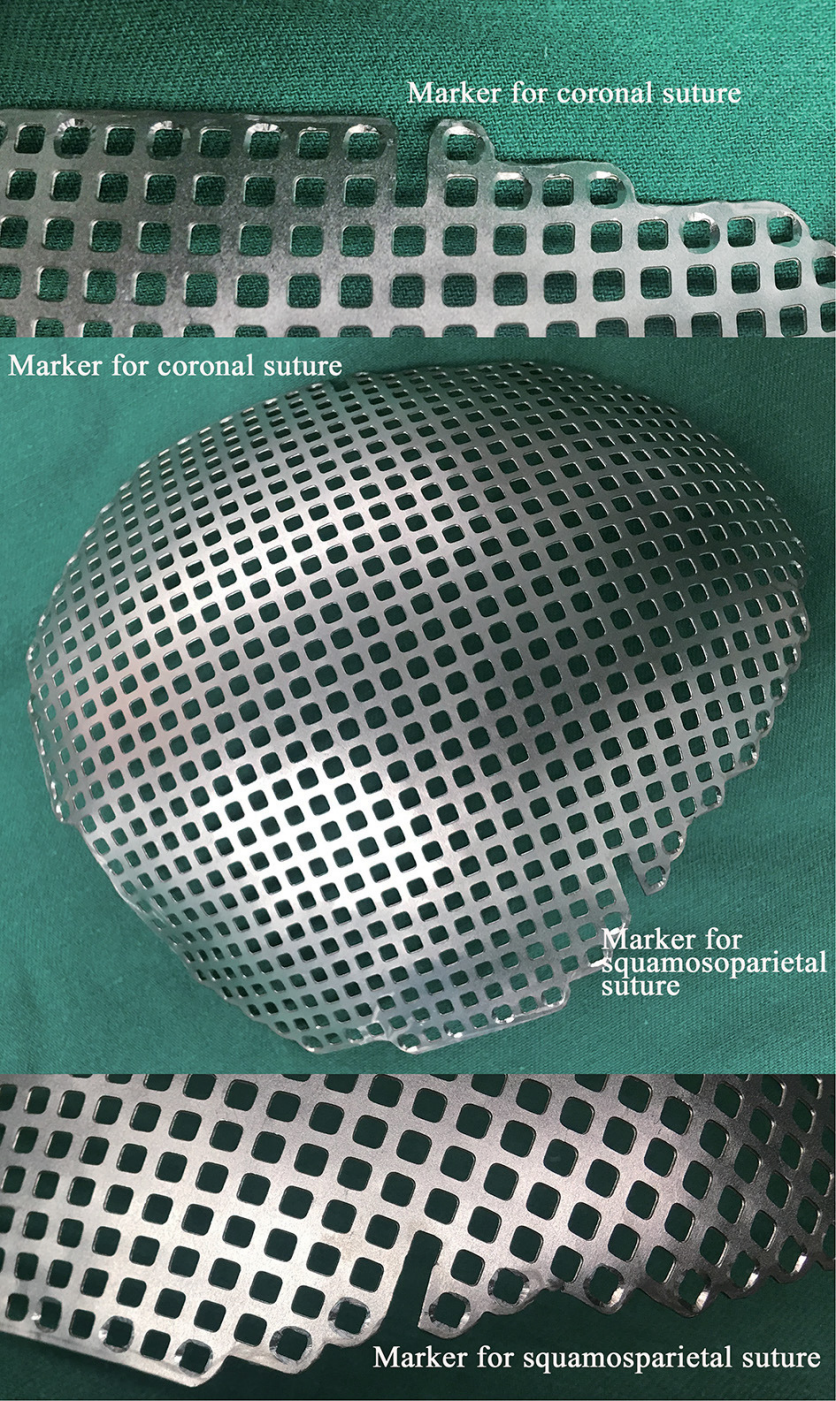
A photograph of a 3D titanium mesh with the anatomical positing markers. 3D, three-dimensional.

We modified the Ti-CP technique and developed a novel technique of marking the coronal and squamosoparietal sutures in 3D titanium mesh. In details, after collecting the preoperative fine-cut computed tomography (CT) data of patients, we used Materialise Mimics version 20 (Materialise Co., Leuven, Belgium) to edit digital imaging and communications in medicine (DICOM) files. During the progress of 3D reconstruction, we marked the coronal and squamosoparietal sutures and designed the titanium mesh that matched the shape, size, and radian of the skull defect. In order to completely cover the skull defect, the margin of the titanium mesh should exceed the skull defect (except for the middle fossa) by about 1 cm. The titanium mesh was trimmed so that it had obvious indentations of coronal and squamosoparietal sutures (~1 cm long) which used as anatomical positioning markers (see Figure 1).

### Study Design

The novel Ti-CP technique was tested on our patient cohort. We designed a prospective, randomized, open-label, single-center trial to verify the effectiveness and safety of this accurate Ti-CP. It was conducted in accordance with the Code of Ethics of the World Medical Association (Declaration of Helsinki) and was approved by the Ethics Committee. All participants or their representatives provided written consent before they participated in the study. We prospectively analyzed the clinical materials of consecutive patients with skull defect at our institution from January 2018 to December 2018, with follow-up, until June 2019. Inclusion criteria of this study were: (1) with a unilateral frontotemporoparietal skull defect following DC and (2) agree to participate in this study. Exclusion criteria were: (1) a documented allergy to titanium, (2) <8 years of age (13), (3) <40 square centimeters of skull defect, (4) non-initial CP, (5) with operative contraindications and not suitable for surgery, and (6) underwent other procedures combined with CP. According to the patients who met the entry criteria in the last 3 years, we set 50 opaque, sealed, and stapled envelopes containing grouping information and set the two groups in a 1:1 ratio. When patients were recruited as volunteers, they were randomly assigned one of these envelopes and hence, they were randomly assigned into the two groups. The corresponding envelopes that marked with the numbers of the patients were opened only after the patients completed all baseline assessments. It was impossible to blind the surgeons because the implanted titanium meshes were easily and clearly identified. In order to ensure the quality of the study, blinding was ensured for the assessors involved in this study. Baseline characteristics, surgical details, postoperative complications, and outcomes were studied and compared. The primary endpoint was postoperative complications occurring at any time within the 6 months after CP. The secondary endpoints included the following: (1) surgery accuracy, (2) visual analog scale for cosmesis (VASC), and (3) Karnofsky performance status (KPS) score at the 6 months after CP.

### Clinical Management

**Figure 2:**
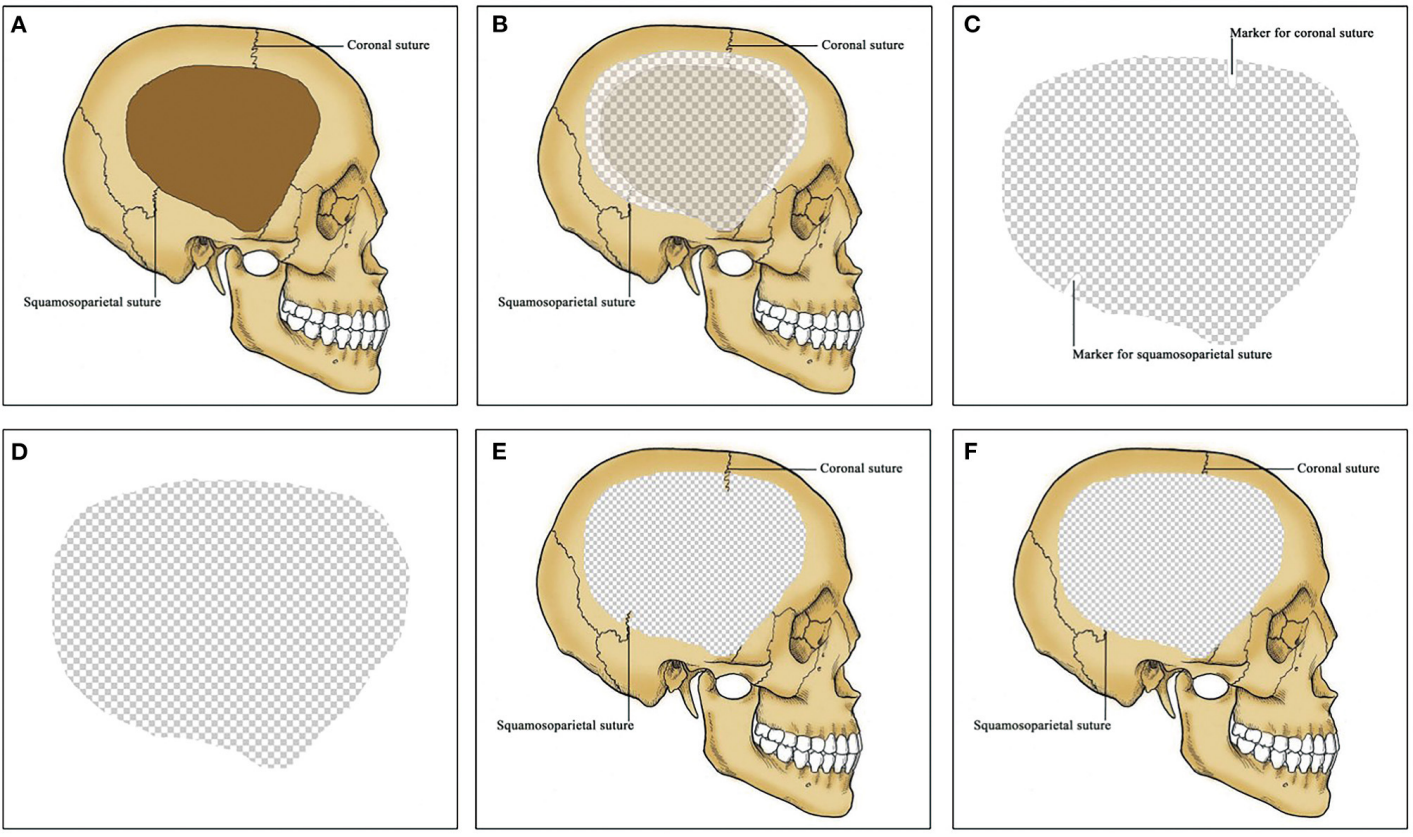
3D titanium mesh for cranioplasty (CP). **(A)** A unilateral frontotemporoparietal skull defect. Coronal and squamosoparietal sutures are observed in the skull. **(B)** CP using 3D titanium mesh. Titanium mesh exceed the skull defect by about 1 cm except for the middle fossa. **(C)** titanium mesh for patients in the intervention group. Coronal and squamosoparietal sutures are marked in the titanium mesh by obvious indentations as anatomical positioning markers. **(D)** titanium mesh for patients in the control group. No anatomical positioning markers in the titanium mesh. **(E)** Titanium cranioplasty (Ti-CP) in the intervention group. The anatomical positioning markers are matched with the coronal and squamosoparietal sutures respectively. **(F)** Ti-CP in the control group. 3D, three-dimensional; CP, cranioplasty; Ti-CP, titanium cranioplasty.

A standardized clinical treatment protocol was used in the management of these patients. The control group was consistent with the intervention group except that there were no anatomical positioning markers in the titanium mesh (see Figure 2). All patients underwent CP under general anesthesia. After aseptic draping, skin incision was made along the previous surgical scar. Subgaleal drain catheter was reserved, and it was removed 24–48 h after surgery. Brain CT for detecting postoperative complications was achieved within 24 h after surgery and during the six-month follow-up. Reoperation was performed when the complications requiring implant removal after CP.

### Data Collection and Definition

A proforma for each patient was used to collect and record the following data: age at the time of CP, gender, medical comorbidities, previous intracranial or incision infection, type of insult, side and size of skull defect, number of surgical procedures pertaining to DC site pre-CP, KPS score at the time of CP, brain bulging classification, surgical details, postoperative complications, and outcomes.

Surgical details included the following data: time between DC and CP, blood loss, operative time, number of screws, and position of the surgeon. Early and delay cranioplasties were defined as less or more than 3 months since DC, respectively (5).

Titanium mesh exposure, surgical-site infection, postoperative hematoma (epidural hematoma or subdural hematoma), postoperative hydrocephalus (or preoperative hydrocephalus worsened), postoperative seizure, and death were defined as major postoperative complications. The implant failure was defined as severe complications after CP as the removal of the implant was necessary.

Outcomes were assessed 6 months after CP in which surgical outcome, cosmetic outcome and neurological outcome was taken into account. Surgical outcome was evaluated by assessors using the surgical accuracy, and it was defined as the similarity between the actual postoperative graft measurements and the surgical plan by the computer using perceptive hash algorithm. Cosmetic outcome was evaluated by patients (or their family members if the patients in a coma) using the VASC. Neurological outcome was assessed by assessors according to the KPS score.

### Statistical Analysis

Data collection and statistical analysis were performed in the Statistical Package for the Social Sciences (SPSS) version 19.0 (IBM Corp., Armonk, NY, USA). Data were described as mean ± standard deviation (SD) and percentages. An independent sample *t* test was used for continuous variables, and a Chi^2^ test or Fisher's exact test was used for categorical variables. Multivariate logistic regression model was used to assess independent predictors of postoperative complications using the logistic regression method. The surgical accuracy was analyzed as a continuous variable and as a categorical variable using receiver operating characteristic (ROC) curve. For the first multivariable analysis model, surgical accuracy was entered as a continuous variable. For the second multivariable analysis model, surgical accuracy was dichotomized as “ < optimal cutoff value” and “≥ optimal cutoff value”. Variables with *P* <0.10 on univariate analysis were considered as potentially independent variables and were entered into the multivariate analysis model. Variables with *P* > 0.10 on present univariate analysis but were reported as independent variables in literatures were also entered into the multivariate analysis model. ROC area under the curve analysis was performed to test the prediction ability of the model. Adjusted odds ratio (OR) and 95% confidence interval (CI) were calculated. Results with *P* < 0.05 were considered to be statistically significant.

## Results

### Patient Population

During the study period, a total of 75 consecutive patients with skull defect were treated at our institution. Nine patients with skull defect size <40 cm^2^, nine patients with bilateral frontotemporoparietal skull defect, seven patients underwent Ti-CP with other procedures, four patients underwent reoperation of Ti-CP due to titanium mesh removal last time, and two patients were <8 years old. Therefore, according to the inclusion criteria described above, a total of 44 patients were enrolled in this study.

### Patient Characteristics

Demographic and baseline characteristics are presented in Table 1. Forty-four patients were included in this study, including 28 (64%) males and 16 (36%) females. The mean age was 44.8 ± 15.2 years (range, 13–75 years). By random assignment, both intervention and control group have 22 patients, and there was no significant difference (*P* > 0.05) in baseline characteristics in the two groups.

**Table 1:** Baseline characteristics of patients.

**Characteristics**	**Total**	**Intervention group**	**Control group**	***P* value**
No. of patients	44	22	22	
Age, year				0.88
Mean ± SD	44.8 ± 15.2	44.5 ± 15.1	45.1 ± 15.8	
Gender, *N* (%)				0.99
Male	28 (64)	14 (64)	14 (64)	
Female	16 (36)	8 (36)	8 (36)	
Hypertension, *N* (%)	12 (27)	6 (27)	6 (27)	0.99
Diabetes, *N* (%)	3 (7)	1 (5)	2 (9)	0.99
Hydrocephalus, *N* (%)	9 (21)	5 (23)	4 (18)	0.99
VPS before CP, *N* (%)	3 (7)	1 (5)	2 (9)	0.99
Subdural fluid collections, *N* (%)	6 (14)	3 (14)	3 (14)	0.99
Epilepsy, *N* (%)	3 (7)	1 (5)	2 (9)	0.99
Previous intracranial or incision infection, *N* (%)	0 (0)	0 (0)	0 (0)	-
Type of insult, *N* (%)				0.55
Trauma	24 (55)	13 (59)	11 (50)	
Stroke	20 (45)	9 (41)	11 (50)	
Skull defect side				0.99
Left	24 (55)	12 (55)	12 (55)	
Right	20 (45)	10 (45)	10 (45)	
Skull defect size, cm^2^				0.30
Mean ± SD	85.9 ± 12.1	87.8 ± 12.9	83.9 ± 11.2	
No. of surgical procedures, *N* (%)				0.99
1	28 (64)	14 (64)	14 (64)	
More than 1	16 (36)	8 (36)	8 (36)	
KPS score at the time of CP				0.26
Mean ± SD	54.6 ± 31.7	60.0 ± 32.8	49.1 ± 30.4	
Brain bulging classification, *N* (%)				0.76
Concave curved	23 (52)	11 (50)	12 (55)	
Flaccid/Tense	21 (48)	11 (50)	10 (45)	

*SD, standard deviation; N, number; VPS, ventriculo-peritoneal shunt; CP, cranioplasty; KPS, Karnofsky performance status*.

### Surgical Details

The surgical details are displayed in Table 2. The mean time between DC and CP was 119.9 ± 49.5 days (range, 80–300 days). Fifteen (34%) patients underwent early surgery. The blood loss was less in the intervention group without significant difference (*P* > 0.05). The operative time, the number of screws used, and the surgeon positions showed no difference in these two groups.

**Table 2:** Surgical details of patients.

**Surgical details**	**Total**	**Intervention**	**Control**	***P* value**
		**group**	**group**	
Time between DC and CP, days				
Mean ± SD	119.9 ± 49.5	128.2 ± 50.4	111.5 ± 48.3	0.27
Early surgery, *N* (%)	15 (34)	5 (23)	10 (45)	0.11
Blood loss, mL				0.19
Mean ± SD	192.1 ± 108.9	170.5 ± 94.7	213.6 ± 119.7	
Operative time, minutes				0.98
Mean ± SD	169.8 ± 40.1	169.9 ± 35.2	169.6 ± 45.3	
No. of screws used				0.85
Mean ± SD	10.8 ± 2.3	10.7 ± 12.2	10.9 ± 2.4	
Position of the surgeon, *N* (%)				0.52
Resident	14 (32)	6 (27)	8 (36)	
Attending	30 (68)	16 (73)	14 (64)	

*DC, decompressive craniectomy; CP, cranioplasty; SD, standard deviation; N, number*.

### Postoperative Complications

Major postoperative complications are shown in Table 3. Complications were noted in 32% (*n* = 14) of the patients, to be more precise, four patients with epidural hematoma in intervention group, six patients with epidural hematoma, two patients with surgical-site infection, one patient with epidural hematoma and surgical-site infection, and one patient with epidural hematoma and postoperative seizure in control group. The implant failure rate was 7% (*n* = 3). The mortality rate was 0. The most common complication was epidural hematoma (*n* = 12), and it was seen in 18% (*n* = 4) in intervention group and 36% (*n* = 8) in control group for no need of surgical revision, without a significant difference (*P* = 0.18). Surgical-site infection was seen in 14% (*n* = 3) of the patients, and these patients were all in the control group and underwent reoperation to remove the titanium mesh.

**Table 3:** Postoperative complications of patients.

**Postoperative**	**Total**	**Intervention**	**Control**	***P* value**
**complications**		**group**	**group**	
Overall complications	14 (32)	4 (18)	10 (45)	0.05
Surgical-site infection, *N* (%)	3 (7)	0 (0)	3 (14)	0.23
Epidural hematoma, *N* (%)	1 2(27)	4 (18)	8 (36)	0.18
Postoperative seizure, *N* (%)	1 (2)	0(0)	1 (5)	0.99
Death, *N* (%)	0 (0)	0 (0)	0 (0)	-
Others, *N* (%)	0 (0)	0 (0)	0 (0)	-
Implant failure, *N* (%)	3 (7)	0 (0)	3 (14)	0.23

*N, number; CP, cranioplasty*.

### Outcomes

Surgical outcome was achieved with a mean surgical accuracy of 95.9%. Most actual postoperative grafts were located posterior and upper than the planned. The mean surgical accuracy in the intervention group (97.8%) was significantly (*P* < 0.001) higher than the control group (94.0%).

Cosmetic outcome was achieved with a mean VASC 7.7. The mean VASC at 6-month follow-up in the intervention group (8.4) was significantly (*P* < 0.001) higher than the control group (7.0).

The mean KPS score at 6-month follow-up was 67.7 in the intervention group and 55.9 in the control group, without a significant difference (*P* = 0.25) (see Table 4).

**Table 4:** Outcomes at 6-month follow-up for patients.

**Outcomes**	**Total**	**Intervention**	**Control**	***P* value**
		**group**	**group**	
Surgical accuracy, %				<0.001
Mean ± SD	95.9 ± 2.7	97.8 ± 1.4	94.0 ± 32.4	
VASC				<0.001
Mean ± SD	7.7 ± 1.0	8.4 ± 0.8	7.0 ± 0.8	
KPS score				0.25
Mean ± SD	61.8 ± 33.9	67.7 ± 32.7	55.9 ± 34.9	

*SD, standard deviation; VASC, visual analog scale for cosmesis; KPS, Karnofsky performance status*.

### Independent Predictors of Overall Postoperative Complications

Fourteen patients with postoperative complications were into the complication (+) group, while others were into the complication (–) group. Results of the univariate analysis for overall postoperative complications at the 6-month follow-up after Ti-CP are showed in Table 5. The univariate analysis indicated that significant differences were detected in hydrocephalus (*P* < 0.05) and surgical accuracy (*P* < 0.001). There were no significant statistical differences in age, type of insult, brain bulging classification, time between DC and CP, operative time, and position of surgeon between the two groups (*P* > 0.05).

**Table 5:** Univariate analysis for overall postoperative complications.

**Characteristics**	**Complication**	**Complication**	***P* value**
	**(−)**	**(+)**	
No. of patients	30	14	
Age, year			0.53
Mean ± SD	43.8 ± 15.7	46.9 ± 14.6	
Gender, *N* (%)			0.95
Male	19 (63)	9 (64)	
Female	11 (37)	5 (36)	
Hypertension, *N* (%)	9 (30)	3 (21)	0.72
Diabetes, *N* (%)	2 (7)	1 (7)	0.99
Hydrocephalus, *N* (%)	9 (30)	0 (0)	0.04
Type of insult, *N* (%)			0.29
Trauma	18 (60)	6 (43)	
Stroke	12 (40)	8 (57)	
KPS score at the time of CP			0.52
Mean ± SD	56.7 ± 32.8	50.0 ± 29.9	
Brain bulging classification, *N* (%)			0.08
Concave curved	13 (43)	10 (71)	
Flaccid/Tense	17 (57)	4 (29)	
Time between DC and CP, days			
Mean ± SD	127.3 ± 55.0	103.9 ± 30.7	0.15
Early surgery, *N* (%)	9 (30)	6 (43)	0.50
Blood loss, mL			0.34
Mean ± SD	203.3 ± 121.7	167.9 ± 72.3	
Operative time, minutes			0.28
Mean ± SD	173.5 ± 42.5	161.6 ± 34.4	
Position of the surgeon, *N* (%)			0.16
Resident	12 (40)	2 (14)	
Attending	18 (60)	12 (86)	
Surgical accuracy, %			<0.001
Mean ± SD	96.9 ± 2.0	93.7 ± 2.7	

*SD, standard deviation; N, number; KPS, Karnofsky performance status; DC, decompressive craniectomy; CP, cranioplasty*.

**Figure 3:**
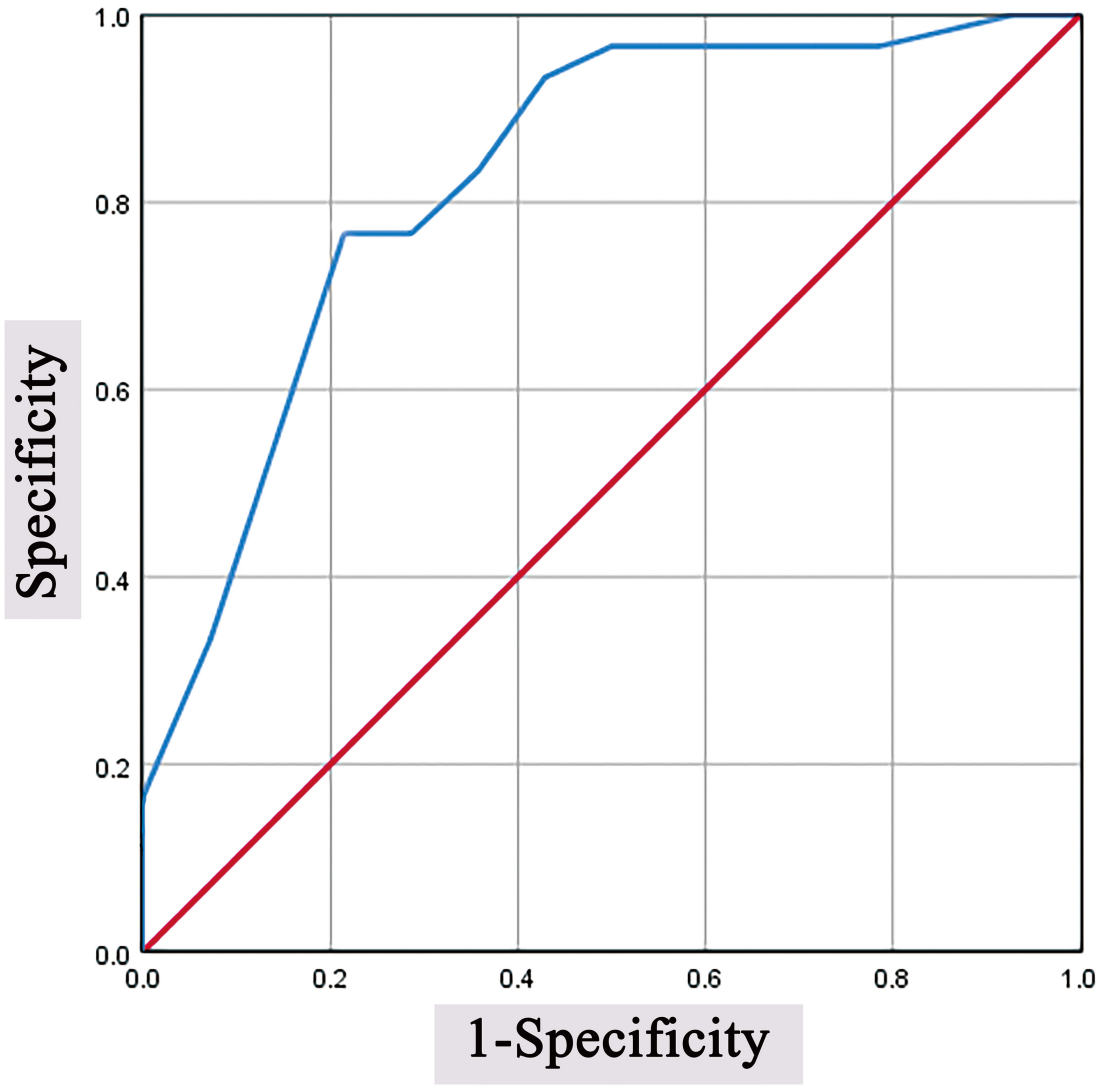
Predictive values of surgical accuracy for overall postoperative complications. The optimal cutoff value was determined as 95.8 in the ROC curve (the sensitivity was 76.7%, and the specificity was 86.7%). ROC, receiver operating characteristic.

The median surgical accuracy in the complication (+) group (93.7) was lower than that in the complication (–) group (96.9). The ROC curve is shown in Figure 3. The optimal cutoff value for surgical accuracy as a predictor for overall postoperative complications was determined as 95.8 in the ROC curve (the sensitivity was 76.7%, and the specificity was 86.7%).

All preoperative variables (hydrocephalus, brain bulging classification and surgical accuracy) with a significance level at *P* < 0.10 in the univariate analysis were included in the multivariate logistic regression model. Variables (age, hypertension, type of insult, time between DC and CP and operative time) with *P* > 0.10 on present univariate analysis, but were reported as independent variables in literatures, were also included in the multivariate logistic regression model. After adjustment for potential confounding variables, surgical accuracy of <95.8% remained significantly associated with overall postoperative complications, and the increased risk was 19.20-fold (OR = 19.20, 95% CI = 3.17–116.45, *P* = 0.001). An ROC curve fitted to this model had an area under the curve of 0.87 (95% CI.76–0.98, *P* < 0.001). Surgical accuracy was significantly associated with overall postoperative complications, and the increased risk was 0.57-fold (OR = 0.57, 95% CI = 0.40–0.82, *P* = 0.002). An ROC curve fitted to this model had an area under the curve of.83 (95% CI.70–0.97, *P* < 0.001) (see Table 6).

**Table 6:** Multivariate analysis for predictors of overall postoperative complications.

**Predictors**	**Unadjusted**		**Adjusted**	
	**OR (95% CI)**	***P* value**	**OR (95% CI)**	***P* value**
Surgical accuracy	–	–	0.57 (0.40–0.82)	0.002
Surgical accuracy <95.8%	19.71 (3.53–110.04)	0.001	19.20 (3.17–116.45)	0.001

*OR, odds ratio; CI, confidence interval*.

## Discussion

We evaluated variation in patient characteristics and outcomes between the intervention and control groups in modern neurosurgery for the purpose of preliminarily verifying the effectiveness and safety of an accurate Ti-CP by marking the coronal and squamosoparietal sutures in 3D titanium mesh. In this study, we found that the intervention group had significantly higher surgical accuracy, better cosmetic prognosis, and less postoperative complications. Surgical accuracy was an independent predictor of overall postoperative complications. Ti-CP with a surgical accuracy more than 95.8% may reduce postoperative complications effectively.

It has been documented that CP was performed by the Incas many centuries ago and first reported by Job Janszoon van Meekeren in 1668 (14, 15). An increasing number of CP procedures have been performed in recent years due to the rising popularity of DC to manage of intracranial pressure for brain trauma and stroke (16). The recovery of neurological deficits following CP is well known (17–24) while severe complications may lead to poor prognosis, even death. Nowadays, neurosurgery has entered the precision era, and the surgical accuracy is getting higher and higher in most procedures of neurosurgery. However, surgical techniques of CP are not accurate. More and more people recognize that CP is not only a structure reconstruction operation but also a cosmetic procedure (25). Many measures are used to achieve good cosmetic outcomes, including 3D shaping of the titanium mesh. The purpose of 3D shaping is to manufacture a titanium mesh that matches the shape, size, and radian of the skull defect. Therefore, the titanium mesh and skull defect theoretically have only one matching location. However, there are no marks on the titanium mesh to accurately match the skull. One of the recent studies indicated that the use of computer-aided design or computer-aided manufacturing for CP improves contour accuracy in the reconstruction of complex skull defects with minimal complications (26). In our study, surgical accuracy <95.8% was the only independent predictor of overall postoperative complications. All of the patients in the intervention group achieved a surgical accuracy more than 95.8%, while only a few patients in the control group achieved the same result. In addition, the intervention group also has better cosmetic outcome. Therefore, we also emphasize the importance of surgical accuracy in Ti-CP. Many junior surgeons achieved higher surgical accuracy than senior surgeons through this novel technique, suggesting that making anatomical positioning markers in the titanium mesh for CP is a simple but effective solution to increase the surgical accuracy for Ti-CP. Given the novelty, simplicity, and feasibility of this idea for improving surgical accuracy in CP, this technique of Ti-CP could bring values and benefits for both surgeons and patients and the statistical changes in accuracy demonstrated here could carry over to clinical value.

The surgical accuracy was 94.0 ± 32.4 in the control group and 97.8 ± 1.4 in the intervention group. It showed that the novel Ti-CP technique improved surgical accuracy by 3.8%. In numerical terms, 3.8% does not seem like much, but it is 3.8 percent close to 100%, so, in a way, it is not easy to raise 3.8%. In addition, by comparing the SD of surgical accuracy between the control group and the intervention group, we found that there was a large difference within the control group, while there was little difference within the intervention group. This indicates that such improvement may eliminate the differences in surgical accuracy caused by subjective factors such as surgical experience so that no matter the level of the surgical experiences of doctors, surgical accuracy can be homogenized and improved. The reasons for the improvement of surgical accuracy and reduction of postoperative complications are considered as follows. Accurate exposure can be achieved with anatomical positioning markers, reducing unnecessary exposure, reducing the risk of wound surface and bleeding, and helping to reduce postoperative epidural hematoma. At the same time, it reduces the number of times of repeatedly placing titanium mesh in the skull to confirm whether the exposure is enough and reduces the chance of infection. In addition, the titanium mesh is a better match to the skull, which can reduce the tension of the incision and reduce the infection caused by poor incision healing.

The surgical accuracy of Ti-CP refers to the accuracy of the placement of titanium mesh. Accurate methods for measuring the surgical accuracy of Ti-CP have not been reported in the literature. We compared the actual postoperative graft measurements with the surgical plan by the computer using perceptive hash algorithm and called this comparison as surgical accuracy. It is a valid tool to be used although it has not been published yet.

The limitations of this study include its relatively small sample size and short time of follow-up. In addition, there was no blinding of surgeons and hence, parameters such as number of screws used may be secondary to bias by the surgeons. However, patients were randomly grouped, and blinding was ensured for the assessors to increase the quality of the study.

## Conclusions

Based on the early experience, making the coronal and squamosoparietal sutures in 3D constructed titanium mesh to be anatomical positioning markers for repairing frontotemporoparietal skull defects increases the surgical accuracy, and produces suitable repair results for patients. Hence, it is a safe and effective improvement for CP. Further studies with larger case series and longer follow-up are needed.

## Data Availability Statement

The original contributions presented in the study are included in the article/Supplementary Material, further inquiries can be directed to the corresponding author/s.

## Ethics Statement

The studies involving human participants were reviewed and approved by the Ethics Committee of the First Affiliated Hospital of Fujian Medical University. Written informed consent to participate in this study was provided by the patients/participants or patients/participants' legal guardian/next of kin.

## Author Contributions

B-SX, F-YW, and S-FZ conceived the manuscript and revised the draft. B-SX wrote the first draft. Y-XL and D-ZK critical revision of manuscript for intellectual content. W-HF implemented analysis and interpretation of data and study supervision. All authors contributed to the article and approved the submitted version.

## Conflict of Interest

The authors declare that the research was conducted in the absence of any commercial or financial relationships that could be construed as a potential conflict of interest.

## Publisher's Note

All claims expressed in this article are solely those of the authors and do not necessarily represent those of their affiliated organizations, or those of the publisher, the editors and the reviewers. Any product that may be evaluated in this article, or claim that may be made by its manufacturer, is not guaranteed or endorsed by the publisher.

## Abbreviations

CP: cranioplasty
DC: decompressive craniectomy
3D: three-dimensional
Ti-CP: titanium cranioplasty
CT: computed tomography
DICOM: digital imaging and communications in medicine
VASC: visual analog scale for cosmesis
KPS: Karnofsky performance status
SD: standard deviation
ROC: receiver operating characteristic
OR: odds ratio
CI: confidence interval
N: number.
