# Supplementary figures and images for: A Novel Titanium Cranioplasty Technique of Marking the Coronal and Squamosoparietal Sutures in Three-Dimensional Titanium Mesh as Anatomical Positioning Markers to Increase the Surgical Accuracy and Reduce Postoperative Complications
Source: Front Surg. 2021 Dec 14;8:754466. doi: 10.3389/fsurg.2021.754466 (PMC8712424; doi:10.3389/fsurg.2021.754466)

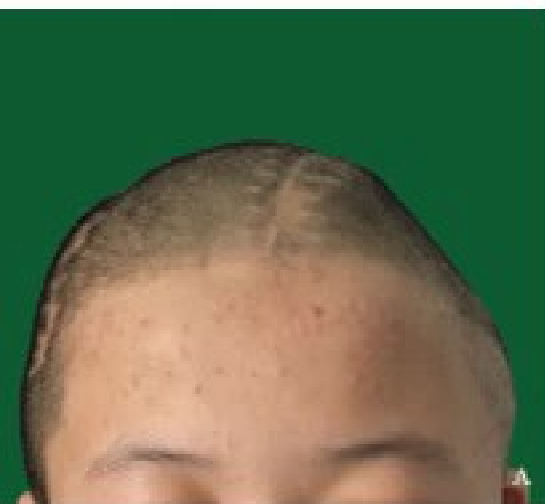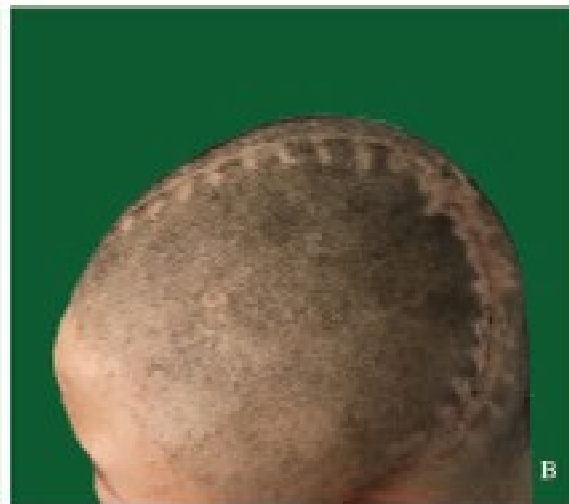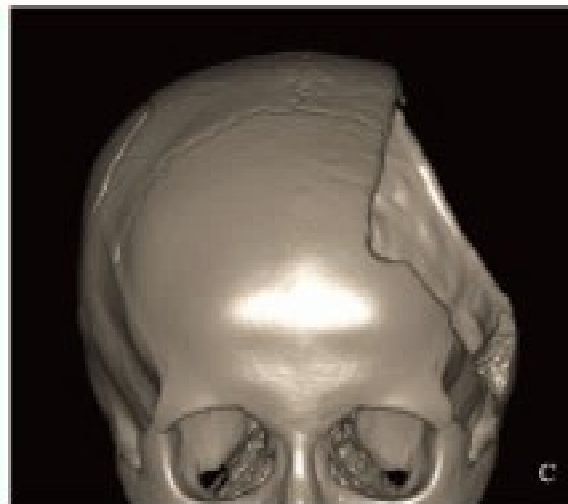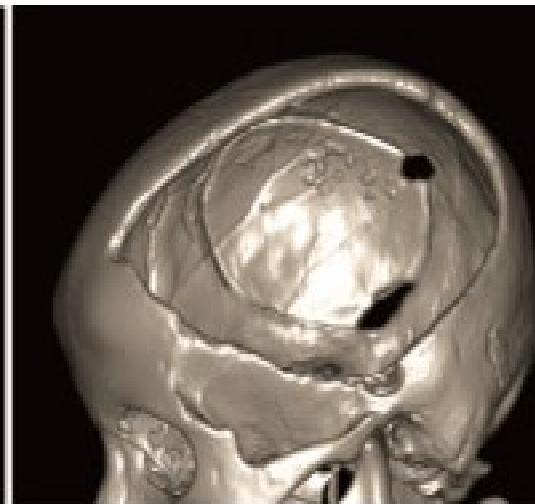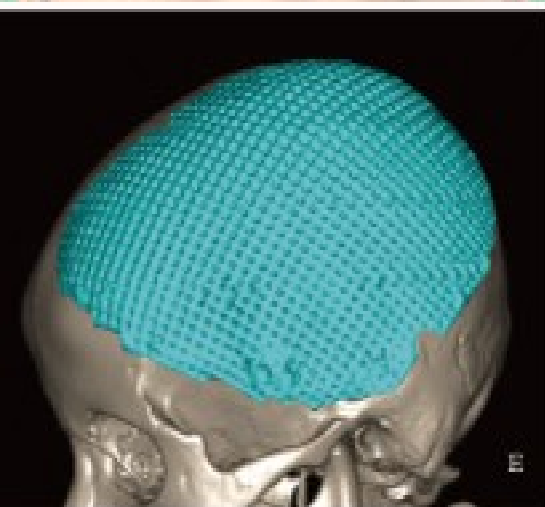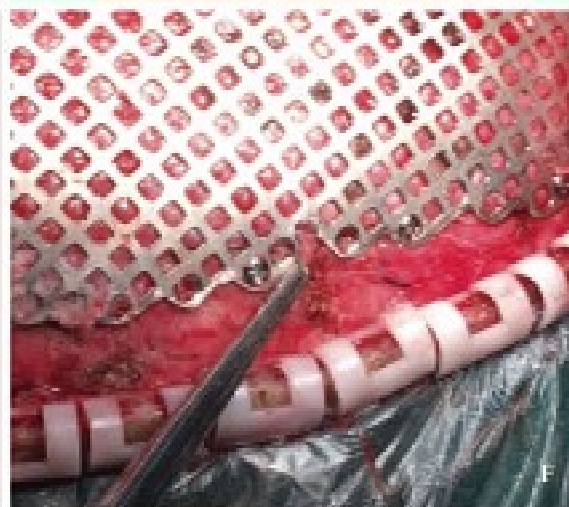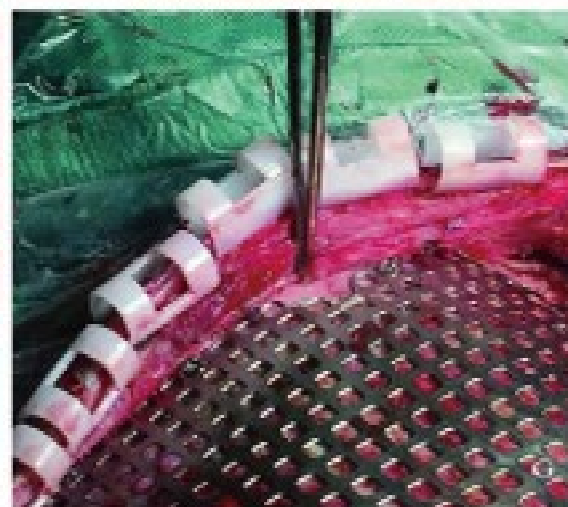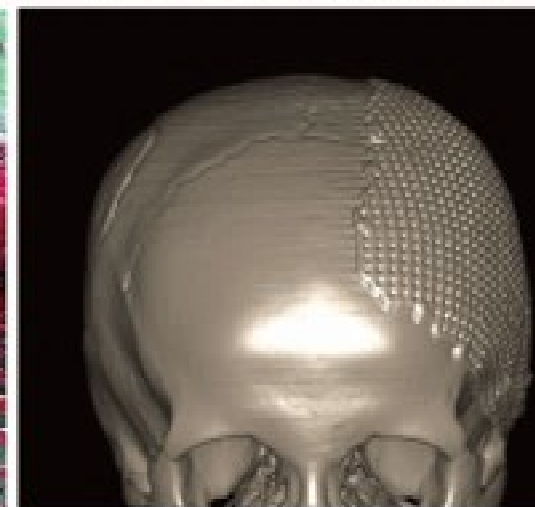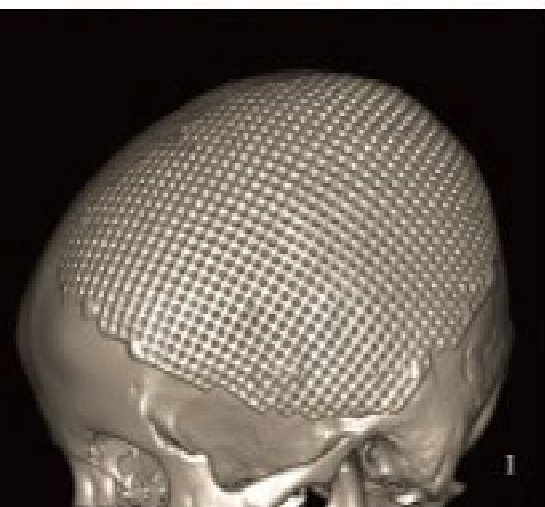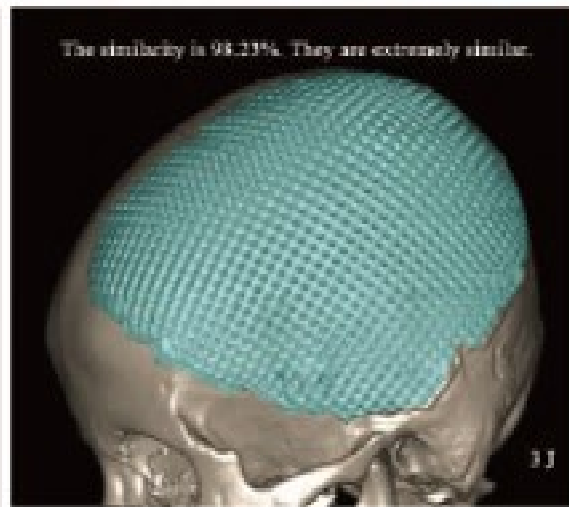

The similarity is 98.23%. They are extremely similar.

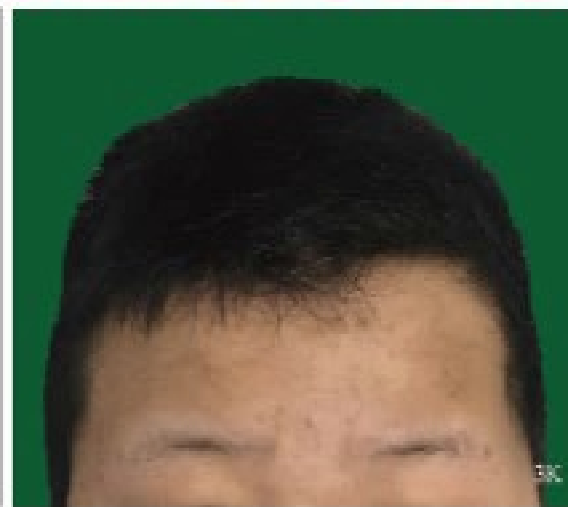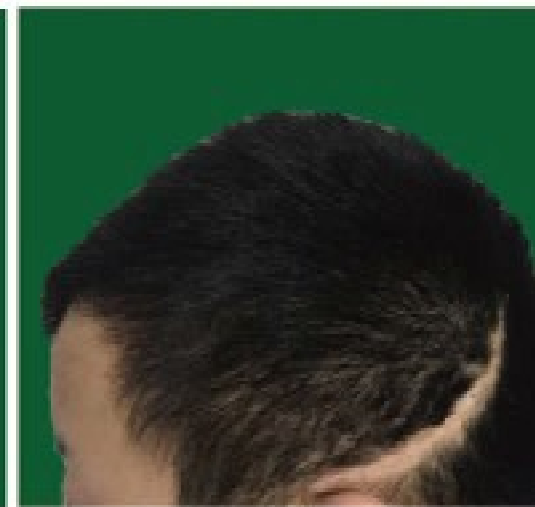

Supplement: Supplementary file 1 [file Data_Sheet_1.PDF]
